# Divergent selection and genetic structure of *Sideritis scardica* populations from southern Balkan Peninsula as revealed by AFLP fingerprinting

**DOI:** 10.1038/s41598-019-49097-x

**Published:** 2019-09-04

**Authors:** Martina Grdiša, Ivan Radosavljević, Zlatko Liber, Gjoshe Stefkov, Parthenopi Ralli, Paschalina S. Chatzopoulou, Klaudija Carović-Stanko, Zlatko Šatović

**Affiliations:** 1 University of Zagreb, Faculty of Agriculture, Department of Seed Science and Technology Svetošimunska 25, 10000 Zagreb, Croatia; 2 Centre of Excellence for Biodiversity and Molecular Plant Breeding (CoE CroP-BioDiv), Svetošimunska 25, 10000 Zagreb, Croatia; 3 University of Zagreb, Faculty of Science, Department of Biology, Division of Botany, Marulićev trg 9A, 10000 Zagreb, Croatia; 4 University Ss. Cyril and Methodius Skopje, Faculty of Pharmacy, Vodnjanska 17, 1000 Skopje, Republic of North Macedonia; 5 Hellenic Agricultural Organization DEMETER, Institute of Breeding and Plant Genetic Resources, Thermi - Thessalonikis, PO Box 60411, 57001 Thessaloniki, Greece

**Keywords:** Genetic markers, Structural variation

## Abstract

*Sideritis scardica* Giseb. is a subalpine/alpine plant species endemic to the central part of the Balkan Peninsula. In this study, we combined Amplified Fragment Length Polymorphism (AFLP) and environmental data to examine the adaptive genetic variations in *S*. *scardica* natural populations sampled in contrasting environments. A total of 226 AFLP loci were genotyped in 166 individuals from nine populations. The results demonstrated low gene diversity, ranging from 0.095 to 0.133 and significant genetic differentiation ranging from 0.115 to 0.408. Seven genetic clusters were revealed by Bayesian clustering methods as well as by Discriminant Analysis of Principal Components and each population formed its respective cluster. The exception were populations P02 Mt. Shara and P07 Mt. Vermio, that were admixed between two clusters. Both landscape genetic methods Mcheza and BayeScan identified a total of seven (3.10%) markers exhibiting higher levels of genetic differentiation among populations. The spatial analysis method Samβada detected 50 individual markers (22.12%) associated with bioclimatic variables, among them seven were identified by both Mcheza and BayeScan as being under directional selection. Four bioclimatic variables associated with five out of seven outliers were related to precipitation, suggesting that this variable is the key factor affecting the adaptive variation of *S. scardica*.

## Introduction

Adaptive genetic variation evolves as a result of changing environmental conditions imposing selective pressure on individuals living in heterogeneous habitats^1^. Elucidating the genetic basis of species adaptations to their environments, the nature of selection imposed by environmental changes and the capacity of a species to respond by evolutionary adaptation^2,3^ is one of the challenging tasks of evolutionary biology. Such information may direct conservation strategies for the long-term persistence of species, especially for those facing rapid climate changes and are threatened with extinction and population decline.

The potential of species adaptation to rapidly changing environments might be assessed by detecting genetic signatures of adaptation to prevailing or historic environmental conditions^4^. Genotyping of numerous random loci across entire genomes enables distinguishing the effects of the evolutionary forces that act on the entire genome (e.g., demographic events, inbreeding or bottleneck) from those influencing only individual loci (e.g., natural selection)^5–7^. Loci that are characterized by higher than expected levels of genetic differentiation are referred to as outlier loci or candidate loci and are considered to be under strong natural selection^7^. Such loci that are exposed to divergent selection pressures across studied populations can be recognized by a higher pairwise index of genetic differentiation values (*F*_*ST*_) if compared to the genome-wide pairwise-*F*_*ST*_^8^. In non-model organisms with a large number of individuals included in a study, Amplified Fragment Length Polymorphism (AFLP) technique has been frequently used in search of such genome regions under selection, when no prior knowledge of their position or importance exists^9,10^. For AFLP genome scans, two analyses methods are most commonly applied: the frequentist method of Beaumont and Nichols^11^ as implemented in LOSITAN^12^ and Mcheza^13^ programs and the hierarchical Bayesian method as implemented in BayeScan program^14^. Between the two methods, it has been suggested that BayeScan is more efficient in recognition of outlier loci with low false positive rate^15^. In addition, by using both geo-referenced environmental data and individual molecular genetic data, a locus-based approach as implemented in Samβada^16^ is often used^17–20^. This approach correlates individual loci occurrence with environmental variables, thus aiming to determine whether each investigated molecular marker is selected by one or a set of specific environmental variables^16^. The described methods provide the opportunity to identify adaptive genetic variation in non-model organisms for which the genomic data are scarce and species are of conservation concern^21^.

Various investigations have been conducted in order to determine the genetic variation of different plant species in relation to the landscape, e. g. *Arabis alpina* L.^22,23^, *Gentiana nivalis* L.^24^, alpine plant species^25^, *Eruca sativa* Mill.^26^, *Keteleeria davidiana* var. *formosana* (Hayata) Hayata^27^, *Cotinus coggygria* Scop.^28^, *Picea abies* (L.) H. Karst.^29^, *Liriodendron chinense* (Hemsl.) Sarg.^30^, *Geropogon hybridus* (L.) Sch. Bip.^21^, *Diplotaxis hara* (Forssk.) Boiss.^31^, etc. Nosil *et al*.^32^ in the literature review state that the available studies usually report 5–10% of loci to be outliers, while Strasburg *et al*.^33^ reported that the proportion of loci identified as outliers ranges from 0.4 to 35.5% with an average value of 8.9%. However, both studies pointed out that the results from different investigations are difficult to compare, as they differ in methodologies used and significant thresholds chosen.

Manel *et al*.^25^ suggested that genetic variation that appears to be caused by natural selection might be the results of isolation by distance, which limits gene flow among populations or the result of secondary contact of populations that survived isolated in glacial refugia. Past historic climatic oscillations have had a considerable impact on present-day biota^34–37^. Numerous phylogeographical investigations carried out over the recent years, have confirmed the latter, elucidating the impact of both ancient and recent history on current European flora and fauna distribution patterns and of genetic variations^38–42^.

*Sideritis scardica* Griseb is an outcrossing diploid (2n = 32)^43^ and perennial subalpine/alpine herbaceous plant, endemic to the central parts of the Balkan Peninsula. The species is distributed in southwest parts of Albania, North Macedonia, Bulgaria and Greece^44^. It is a plant of the alpine zone, occurring in dry stony meadows, mainly at altitudes 1600–2300 m a.s.l., and only occasionally down to 500 m a.s.l. on limestone^45,46^. In Greece, most abundant populations of *S*. *scardica* can be found in mountains of the North and Central regions such as Vermion, Voras, Tzena, Paikon, Pangeon, Menikio, Falakro, Mt. Olympus, Mt. Ossa, etc.^46^. In North Macedonia, it occurs in mountains of Central and Western parts of the country^47^, namely in Mt. Shara, Mt. Suva Gora, Mt. Bistra, Mt. Ilina, Mt. Kozuf and Mt. Kajmakchalan.

*Sideritis scardica* has successfully established populations in contrasting environmental conditions and therefore represents an ideal system to examine the effect of specific environmental variables on genetic variation found between the genomes of individuals.

In this study, we selected nine *S*. *scardica* from southern parts of Balkan Peninsula that were exposed to contrasting environmental conditions. Altitudinal and climatic data were used to characterize the environmental conditions at each collecting site. To test our hypothesis that signals of natural selection (i.e. *F*_*ST*_ outlier loci) can be found across chosen *S*. *scardica* populations, we performed AFLP fingerprinting followed by comprehensive analyses of adaptive genetic variation and underlying drivers of adaptive divergence. In addition, possible influences of past climatic oscillations on population genetic structure and diversity of studied populations were addressed.

## Results

### Within population diversity

**Figure 1:**
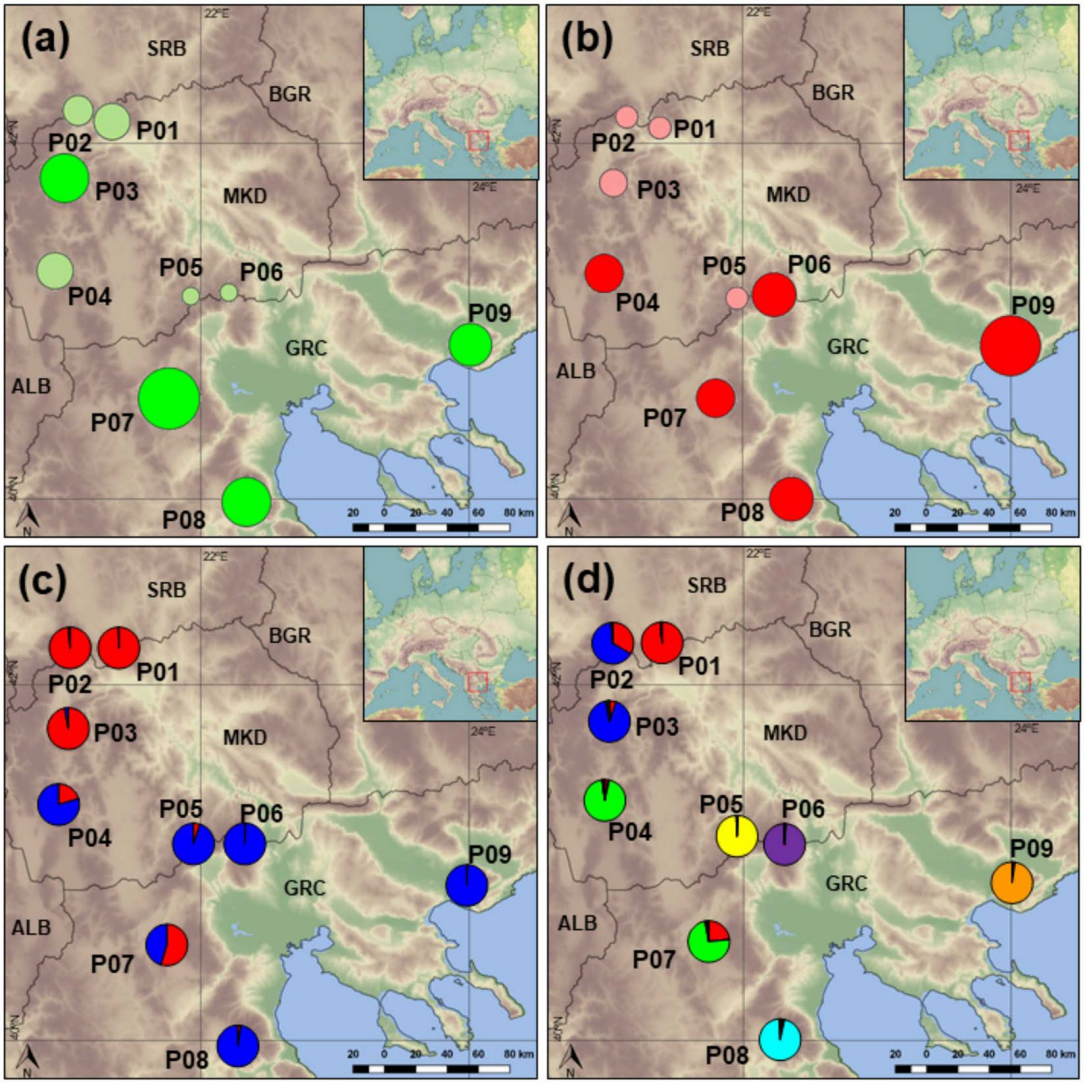
Genetic diversity and structure of 12 *Sideritis scardica* populations from North Macedonia and Greece: Shannon’s information index (**a**), Frequency down-weighted marker values (**b**), Genetic structure derived from Bayesian analysis using STRUCTURE at *K* = 2 (**c**) and *K* = 7 (**d**). In (**a**) and (**b**), the size of the dots is directly proportional to the depicted values.

The proportion of polymorphic markers varied from 25.37% in population P05 Mariovo to 50.00% in P07 Mt. Vermio. Private alleles were detected in all the analyzed populations and P08 Mt. Olympus had the highest number, 15 respectively. Shannon’s information index (*I*) indicated that the total diversity (*H*_*t*_) was 0.417 with average intrapopulation diversity (*H*_*p*_) being 0.238. Similar levels of diversity within populations (*H*_*p*_*/H*_*t*_) (57.20%) and among populations (42.80%) were determined. Shannon’s index per population ranged from 0.175 (P05 Mariovo) to 0.305 (P07 Mt. Vermio) (Fig. 1a). Estimates of frequency down-weighted marker values (*DW*) were above average for five populations (P04, P06-P09) (Fig. 1b). Expected heterozygosity (*H*_*E*_) ranged from 0.095 (P06 Mt Kozuf) to 0.133 (P07 Mt. Vermio) (Table 1).

**Table 1:** Sampling localities and molecular diversity revealed by AFLP markers in nine *Sideritis scardica* populations from North Macedonia and Greece.

No.	Country	Sampling locality	Elevation (m a.s.l.)	*n*	*P%*	*N*_*pr*_	*I*	*H*_*E*_	*DW*
P01	MKD	Mt. Skopska Crna Gora	641	13	30.35	3	0.203	0.111	69.54
P02	MKD	Mt. Shara	1682	19	34.58	3	0.226	0.109	64.18
P03	MKD	Mt. Suva Gora	1558	18	37.56	4	0.245	0.121	88.42
P04	MKD	Mt. Ilinska	1663	17	43.53	11	0.257	0.113	128.47
P05	MKD	Mariovo	965	14	25.37	4	0.175	0.098	73.59
P06	MKD	Mt. Kozuf	1455	21	32.59	8	0.200	0.095	135.36
P07	GRC	Mt. Vermio	997	22	50.00	8	0.305	0.133	118.05
P08	GRC	Mt. Olympus	1721	21	45.02	15	0.266	0.123	145.03
P09	GRC	Mt. Paggaio	1400	21	42.54	13	0.266	0.118	201.60

*n* - sample size; *%P* - proportion of polymorphic bands; *N*_*pr*_- number of private bands; *I* - Shannon’s information index; *H*_*E*_ - gene diversity of a population assuming Hardy-Weinberg equilibrium; *DW* - frequency down-weighted marker values.

### Genetic relationships and population structure

The coefficient of genetic differentiation (*F*_*ST*_) ranged from 0.115 between P02 Mt. Shara and P03 Mt. Suva Gora to 0.408 between P01 Mt. Skopska Crna Gora and P06 Mt. Kozuf, with an average value of 0.263. The Analysis of Molecular Variance (AMOVA) further showed that most of the genetic diversity was attributable to differences among individuals within populations (63.25%). However, *φ*_*ST*_ value for the among-populations component was significant (*φ*_*ST*_ = 0.367, *P* < 0.0001) (Table 2), suggesting the existence of population differentiation. Pairwise *φ*_*ST*_ values ranged from 0.202 between P02 Mt. Shara and P03 Mt. Suva Gora to 0.534 between P01 Mt. Skopska Crna Gora and P06 Mt. Kozuf.

**Table 2:** AMOVA analysis for the partitioning of AFLP diversity among and within nine *Sideritis scardica* populations.

Source of variation	df	Variance components	Percentage of variation	*ϕ*-statistics	*P(ϕ*)
Among populations	8	13.22	36.75	0.367	<0.0001
Within populations	517	22.76	63.25		

The Neighbor-joining tree based on Dice’s distance matrix showed that all the individuals belonging to the same population were consistently grouped together (Supplementary Fig. S1). Bootstrap support was strong (>90%) in the case of population clusters P05 Mariovo, P06 Mt. Kozuf, P08 Mt. Olympus and P09 Mt. Paggaio, all sampled in the south-eastern part of the studied area. Bootstrap values were moderate for P04 Mt. Ilinska (81%) and weak for P03 Mt. Suva Gora (57%), while all the other population clusters were not supported, having bootstrap values lower than 50%. The cluster comprised of populations P01 Mt. Skopska Crna Gora, P02 Mt. Shara, P03 Mt. Suva Gora, P04 Mt. Ilinska, and P07 Mt. Vermio showed weak bootstrap support (69%).

Model-based clustering analysis using STRUCTURE revealed the same pattern of grouping as the distance-based method. Average estimates of the likelihood of the data, conditional on a given number of clusters, *ln [Pr(X|K)]*, kept increasing with higher *K* as did the standard deviations among different runs for each *K*. The highest *ΔK* was observed for *K* = 2 (826.03), followed by that of *K* = 7 (9.89) (Supplementary Fig. S2). At *K* = 2, all the individuals belonging to populations sampled in the north-western part of the studied area, i. e. P01 Mt. Skopska Crna Gora, P02 Mt. Shara and P03 Mt. Suva Gora were assigned to cluster A; while cluster B included all the individuals from P05 Mariovo, P06 Mt. Kozuf, P08 Mt. Olympus and P09 Mt Paggaio, sampled in the south-eastern part of the studied area (Fig. 1c). North-western montane population P04 Mt. Ilinska and south-eastern colline population P07 Mt. Vermio were admixed between the two clusters.

At *K* = 7, each population formed its respective cluster, except for the populations P02 Mt. Shara and P07 Mt. Vermio, that were admixed between two clusters (Fig. 1d). Population P02 Mt. Shara was admixed between clusters A consisting of individuals from population P01 Mt. Skopska Crna Gora and B including individuals from population P03 Mt. Suva Gora, while the individuals from population P07 were assigned either to cluster A or to cluster C together with all the individuals from P04 Mt. Ilinska.

BAPS (Bayesian Analysis of Population Structure) mixture analyses with or without spatially informative priors yielded nearly identical results to those obtained by STRUCTURE at *K* = 7 (Supplementary Fig. S3). The best partitions received log marginal likelihoods of -17761,59 at P = 1 (without using geographic coordinates as informative priors) and -17937,18 at P = 1 (spatial clustering). In both cases, each population formed its respective cluster, except for the populations P02 Mt. Shara and P07 Mt. Vermio, that were admixed between two clusters.

**Figure 2:**
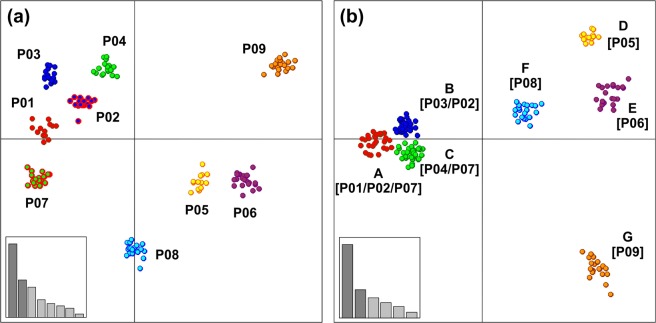
Scatterplot of *Sideritis scardica* samples fromNorth Macedonia and Greece on the two principal components of Discriminant Analysis of Principal Components (DAPC) obtained with prior information of individual population membership (**a**: nine populations) and without that information (**b**: seven clusters). Eigenvalues of the analysis are shown as an inset for each graph with dark gray bars representing those used in the scatterplot.

In both Discriminant Analyses of Principal Components (DAPC) performed with and without prior information of individual population membership we retained 50 principal components representing 77.97% of total genetic variation. The scatterplot of the first analysis (with prior; Fig. 2a) showed that along the first principal component the populations P05 Mariovo, P06 Mt. Kozuf, P08 Mt. Olympus and P09 Mt. Paggaio were separated from the remaining populations, that were positioned close to each other. In the second analysis (without prior) we covered a range of possible clusters from 1 to 10. The lowest BIC value (556) corresponded to *K* = 7 (Supplementary Fig. S4). The clusters A, B and C consisted of individuals from more than one population: cluster A grouped individuals belonging to P01 Mt. Skopska Crna Gora, P02 Mt. Shara and P07 Mt. Vermio, cluster B from P02 Mt. Shara and P03 Mt. Suva Gora, while cluster C grouped individuals from P04 Mt. Ilinska and P07 Mt. Vermio (Fig. 2b). On the other hand, clusters D, E, F and G corresponded completely with the population memberships of the individuals: D (P05 Mariovo), E (P06 Mt. Kozuf), F (P08 Mt. Olympus) and G (P09 Mt. Paggaio). In congruence with the results of model-based clustering analysis using both STRUCTURE (at *K* = 7) and BAPS, the DAPC analysis without prior information of individual population membership revealed that populations P02 Mt. Shara and P07 Mt. Vermio consisted of individuals grouped in more than one cluster, while the rest of the populations belonged to a single genetic cluster.

### Isolation by distance

The Mantel test was significant, and we found a positive correlation between pairwise genetic distances and spatial distances among all nine populations (*r* = 0.397, *P*_*Mantel*_ = 0.017), and 15.7% of the genetic variation could be explained by geographical distances (Supplementary Fig. S5).

### Principal Component Analysis

**Figure 3:**
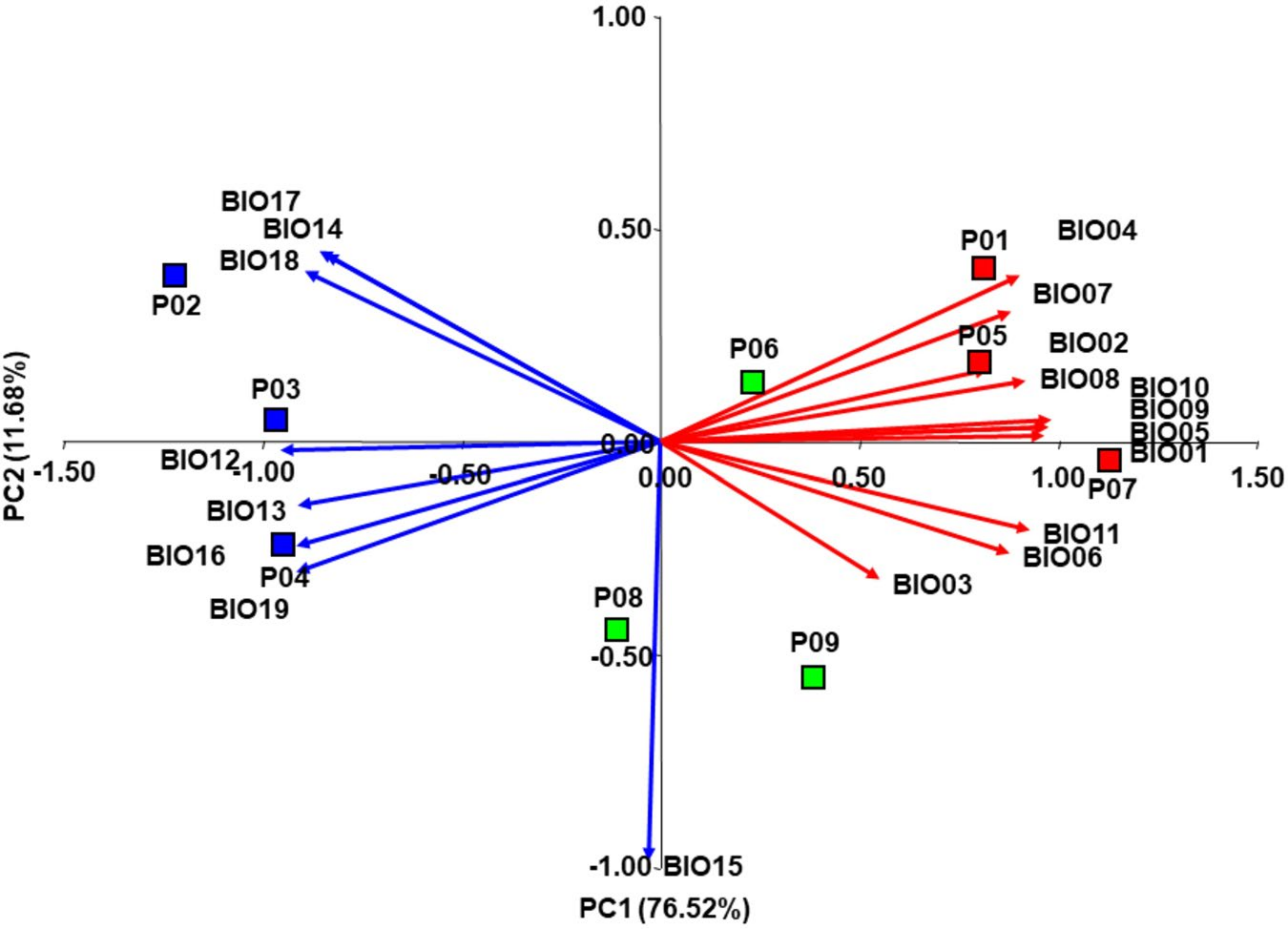
Biplot of Principal Component Analysis (PCA) based on 19 bioclimatic variables of nine *Sideritis scardica* sampling sites. The temperature-related variables (BIO01-BIO11) are shown as red vectors while the precipitation-related variables (BIO12-BIO19) are shown as blue vectors. The colline populations (P01, P05, P07) are shown in red, the north-western montane populations (P02, P03, P04) in blue and the south-eastern montane populations (P06, P08, P09) in green.

The 19 bioclimatic variables used in the study were highly inter-correlated. Out of 171 pairwise examinations, a strong positive correlation (*r* > 0.70) was found in 61 cases, while in 58 cases a strong negative correlation (*r* < −0.70) was identified (Supplementary Table S1). A Principal Component Analysis based on the correlation matrix revealed that the first three principal components had an eigenvalue ˃1, jointly explaining 94.39% of the total variation. Ten temperature-related variables (BIO01, BIO02, BIO04-BIO11) exhibited a strong positive correlation (*r* > 0.70), while seven precipitation-related variables (BIO12-BIO14, BIO016-BIO019) exhibited a strong negative correlation (*r* < −0.70) with the first principal component (PC1) that explained as much as 76.52% of the total variation (Table 3). With the second principal component (PC2), explaining 11.68% of the total variation, only a single variable was strongly negatively correlated [Precipitation Seasonality (BIO15)]. The biplot constructed by the two principal components showing populations and bioclimatic variables (as vectors) is presented in Fig. 3. Along the PC1 the north-western montane populations (P02 Mt. Shara, P03 Mt. Suva Gora and P04 Mt. Ilinska) were clearly separated from colline populations (P01 Mt. Skopska Crna Gora, P05 Mariovo, P07 Mt. Vermio), while south-eastern montane populations (P06 Mt. Kozuf, P08 Mt. Olympus, P09 Mt. Paggaio) were positioned near the center of the plot. The sampling sites of the north-western montane populations were characterized by substantially higher precipitation and lower temperature, in comparison to the sampling sites of colline populations while the sampling sites of south-eastern montane populations displayed the intermediate values. The PC2 separated populations P08 Mt. Olympus and P09 Mt. Paggaio from the rest as those two sampling sites exhibited above-average values of Precipitation Seasonality (BIO15).

**Table 3:** Correlations between 19 environmental variables (BIO01-BIO19) and the first three principal components.

No.	Environmental variable	PC1	PC2	PC3
BIO01	Annual Mean Temperature	0.965^***^	0.015^ns^	0.120^ns^
BIO02	Mean Diurnal Range	0.822^***^	0.170^ns^	−0.533^ns^
BIO03	Isothermality	0.550^ns^	−0.322^ns^	−0.749^ns^
BIO04	Temperature Seasonality	0.903^***^	0.393^ns^	0.016^ns^
BIO05	Max Temperature of Warmest Month	0.984^***^	0.051^ns^	−0.034^ns^
BIO06	Min Temperature of Coldest Month	0.878^***^	−0.260^ns^	0.310^ns^
BIO07	Temperature Annual Range	0.883^***^	0.307^ns^	−0.321^ns^
BIO08	Mean Temperature of Wettest Quarter	0.919^***^	0.142^ns^	0.004^ns^
BIO09	Mean Temperature of Driest Quarter	0.974^***^	0.036^ns^	0.115^ns^
BIO10	Mean Temperature of Warmest Quarter	0.978^***^	0.036^ns^	0.104^ns^
BIO11	Mean Temperature of Coldest Quarter	0.930^***^	−0.205^ns^	0.164^ns^
BIO12	Annual Precipitation	−0.956^***^	−0.018^ns^	−0.103^ns^
BIO13	Precipitation of Wettest Month	−0.913^***^	−0.149^ns^	−0.170^ns^
BIO14	Precipitation of Driest Month	−0.858^***^	0.449^ns^	−0.070^ns^
BIO15	Precipitation Seasonality	−0.028^ns^	−0.984^***^	−0.025^ns^
BIO16	Precipitation of Wettest Quarter	−0.916^***^	−0.244^ns^	−0.089^ns^
BIO17	Precipitation of Driest Quarter	−0.844^***^	0.443^ns^	−0.071^ns^
BIO18	Precipitation of Warmest Quarter	−0.896^***^	0.402^ns^	0.082^ns^
BIO19	Precipitation of Coldest Quarter	−0.918^***^	−0.306^ns^	−0.014^ns^
	Eigenvalue	14.54	2.22	1.18
	% variance	76.52	11.68	6.19

Ns – non-significant; *significant at *P* < 0.05; **Significant at *P* < 0.01; ***Significant at *P* < 0.001.

### Adaptive genetic variation

**Figure 4:**
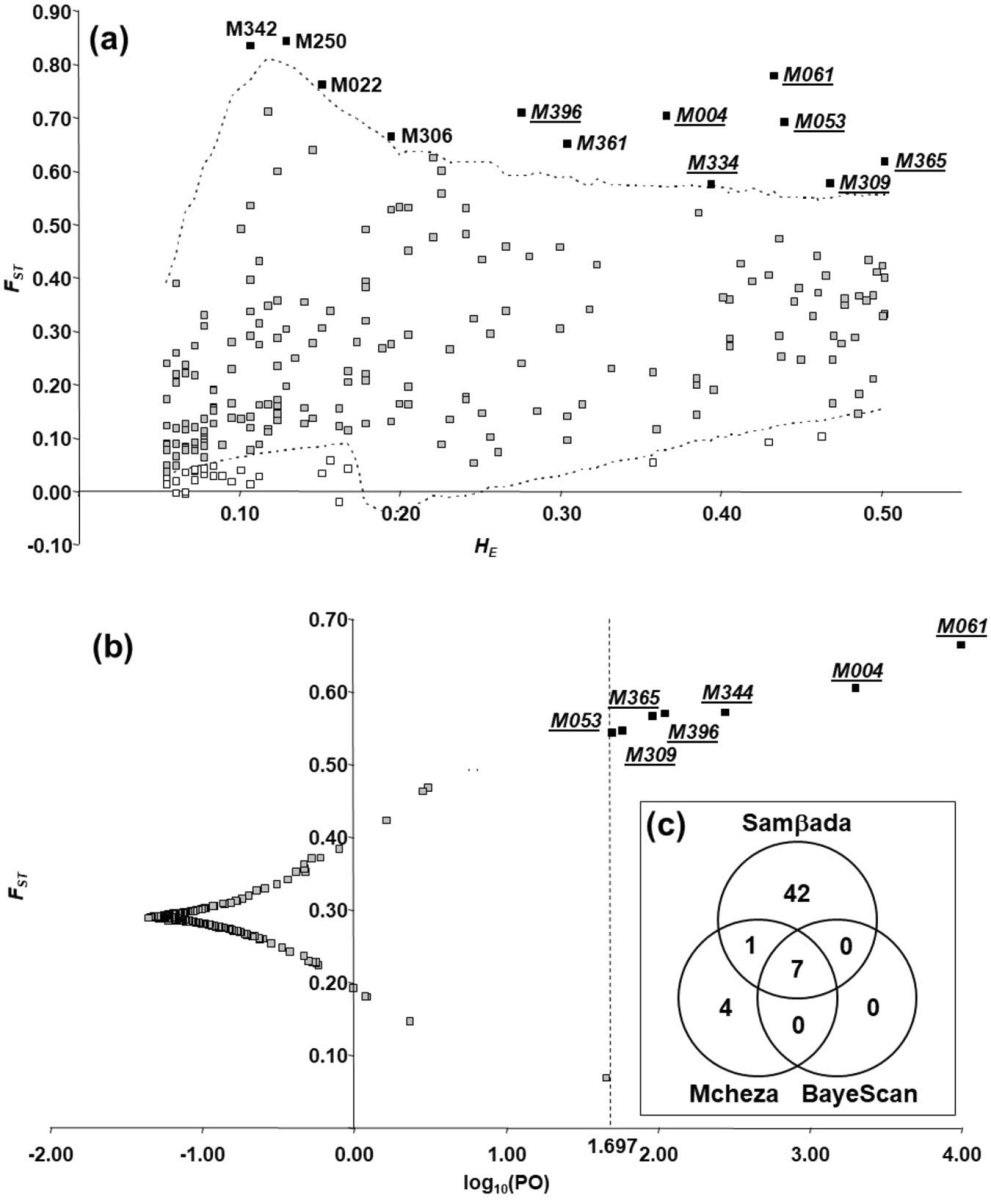
Identification of *F*_*ST*_ outlier loci using (**a**) Mcheza and (**b**) BayeScan, and (**c**) the Venn diagram summarizing the number of loci identified as *F*_*ST*_ outlier loci by Mcheza and BayeScan and significantly associated with environmental variables by Samβada. In (A) *F*_*ST*_ values were plotted against its heterozygosity (*H*_*E*_). The dashed lines represent the 99% confidence intervals. Loci under positive selection are indicated as black dots, those under balancing selection as white dots and neutral as grey dots. Loci under positive selection detected also by BayeScan are underlined while those identified by Samβada are shown in italics. In (B) *F*_*ST*_ values were plotted against the log_10_ of the posterior odds (PO). The vertical line shows the critical PO used for identifying outlier markers [FDR < 0.01; PO = 49.76; log_10_(PO) = 1.697]. Loci under positive selection detected also by Mcheza are underlined while those identified by Samβada are shown in italics.

A total of 226 AFLP markers were used to identify the outlier loci (after the removal of markers with a frequency below 3% or above 97%). With a confidence level set to 99%, Mcheza detected a total of 38 outlier loci (16.81%), possibly under selection, among which 12 (5.31%) are/were under directional and 26 (11.50%) under balancing selection (Fig. 4a). BayeScan identified seven loci (3.10%) exceeding the threshold for very strong evidence of selection [False Discovery Rate (FDR < 0.01; posterior odds (PO) = 37; log_10_ (PO) = 1.697], none of them under stabilizing selection. All seven loci were also detected by Mcheza as outliers (Fig. 4b). After calculating logistic regressions between all possible marker/bioclimatic variable pairs (a total of 4,294 models), Samβada detected 190 (4.42%) significant models involving 50 markers (22.12%) correlated with one up to 17 bioclimatic variables. Bioclimatic variables associated with 15 or more markers were Isothermality (BIO03), Precipitation Seasonality (BIO15), Precipitation of Driest Quarter (BIO17) and Precipitation of Warmest Quarter (BIO18). Out of 50 markers detected by Samβada, seven were identified by both Mcheza and BayeScan (Table 4). Thus, out of a total of 226 markers, seven (3.10%) were identified across three methods as presented in the Venn diagram illustrating the overlap in outlier detection (Fig. 4c). All 19 bioclimatic variables were associated with at least one outlier marker. In general, precipitation-related variables (BIO12-BIO19) were associated with more outlier markers (from one to five) than temperature-related variables (BIO01-BIO11; from one to three). All four bioclimatic variables associated with five out of seven outlier markers were related to precipitation: Annual Precipitation (BIO12), Precipitation of Driest Month (BIO14), Precipitation of Driest Quarter (BIO17) and Precipitation of Warmest Quarter (BIO18).

**Table 4:** Total number of markers associated with each environmental variable as detected using Samβada and the number of markers identified also by both Mcheza and BayeScan as being under directional selection.

No.	Environmental variable	No. of significant markers	
		*Samβada*	BayeScan/Mcheza
BIO01	Annual Mean Temperature	5	1
BIO02	Mean Diurnal Range	10	1
BIO03	Isothermality	15	3
BIO04	Temperature Seasonality	8	1
BIO05	Max Temperature of Warmest Month	7	1
BIO06	Min Temperature of Coldest Month	4	1
BIO07	Temperature Annual Range	8	1
BIO08	Mean Temperature of Wettest Quarter	3	1
BIO09	Mean Temperature of Driest Quarter	6	1
BIO10	Mean Temperature of Warmest Quarter	6	1
BIO11	Mean Temperature of Coldest Quarter	7	2
BIO12	Annual Precipitation	13	5
BIO13	Precipitation of Wettest Month	10	3
BIO14	Precipitation of Driest Month	14	5
BIO15	Precipitation Seasonality	22	3
BIO16	Precipitation of Wettest Quarter	10	3
BIO17	Precipitation of Driest Quarter	16	5
BIO18	Precipitation of Warmest Quarter	19	5
BIO19	Precipitation of Coldest Quarter	7	1

## Discussion

### Genetic diversity and structure

The obtained results demonstrated relatively low within population genetic diversity and significant population differentiation. Within population diversity in terms of polymorphic markers (average 36.21%) and gene diversity (average 0.11) was somewhat higher for the south-eastern *S*. *scardica* populations. However, overall gene diversity levels were lower than the values reported by Nybom^48^ for the endemic plants using dominant markers (*H*_*E*_ = 0.20). Private bands were detected in all analyzed populations, ranging from 3 to 15. Generally, south-eastern populations tend to have more private bands and higher *DW* values. In concordance with detected private bands, genetic differentiation among analyzed populations was significant (*F*_*ST*_ = 0.263), indicating moderate population differentiation. Sampled *S*. *scardica* populations are distributed among fragmented mountain ranges, separated by deep valleys and consequently, pollen and seed dispersal among them is limited. A positive and significant correlation between genetic and geographical distances revealed a pattern of isolation by distance across the distribution range of *S*. *scardica* and further confirmed limited gene flow between analyzed populations, which facilitates the establishment of local adaptations. However, more continuous distribution in the recent past and historical gene flow among analyzed populations is a possibility. Evidence for historical gene flow between neighboring populations of *S*. *scardica* is provided from both distance and model-based methods, i. e. populations with closer proximity to each other are generally also genetically closer. During the Quaternary, suitable habitats for mountain species became limited and less available^40^ and it can be assumed that the species survived by altitudinal shifts to adjacent lowlands, as it has been proposed for other mountain biota^34,49^, followed by postglacial remigration to higher altitudes. Such elevational shifts have been documented for other cold-adapted taxa, i. e. arctic and alpine species which escaped the postglacial warming to cooler climates by altitudinal and/or latitudinal range shifts^50^. Furthermore, it is possible that during these colder periods when the species presumably occupied significantly lower altitudes than today, inter-population hybridization involving different gene pools easily occurred, at least in some areas. As a consequence, in areas where genetically divergent groups of populations came into contact, admixed population-genetic patterns comprising of elements from different gene pools (07 Mt. Vermio) may be observed. During the post-glacial period, remigration to higher altitudes resulted in inter-population isolation. However, this recolonization happened relatively recently and there was not enough time for the development of more pronounced differentiation among populations. Some of the obtained results support this hypothesis, e. g. population P09 Mt. Paggaio, which is geographically isolated from populations P04 Mt. Ilinska, P05 Mariovo and P06 Mt. Kozuf in present days originates from a common ancestral population as revealed by STRUCTURE analysis at *K* = 2. For population P09 Mt. Paggaio, we presume that it survived isolated for a longer period. Our estimates of *DW* values (201.60) and detected number of private alleles (13) support this idea as their higher values are expected in long-term isolated populations where rare markers accumulate due to mutations^41^.

When discussing the detected levels of differentiation, it can also be assumed that because of already discussed climatic oscillations and resulting migrations along the altitudinal gradient, at least some of these populations have experienced severe fluctuations in size and consequently a strong genetic drift. Since the genetic drift, as well as its most extreme form (i.e. the bottleneck), is a stochastic fluctuation in allele frequencies, it could not only cause the loss of the genetic variation, but also increase the amount of genetic differentiation among populations^51,52^. When a population is founded by a limited number of individuals (i.e. through either bottleneck or founder effect), it is expected that its genetic composition may substantially differ from source population and other populations founded through the same processes, and from the identical source population, simply because of stochastic nature of genetic drift^53,54^. Consequently, it is possible to assume that at least some of the detected genetic differentiation originates from genetic drift, followed by the absence of inter-population gene flow, rather than solely natural selection.

Overall, the results implicate that connectivity among populations might have been maximal during the glaciation periods and interrupted in the post-glacial period with remigration to higher altitudes, resulting in significant and moderate levels of genetic differentiation among populations.

### Adaptive divergence

A total of 226 AFLP markers were analyzed to find the signals of divergent selection among nine natural *S*. *scardica* populations sampled in environments reflecting variation in altitude, precipitation and temperature, by performing widely used *F*_*ST*_*-*outlier methods Mcheza and BayeScan, and a genotype-environment association analysis with Samβada. Mcheza detects loci with unusually high or low *F*_*ST*_ values using the frequentist method, while BayeScan uses the Bayesian approach and directly estimates the posterior probability that a given locus is under selection by defining two alternative models (with and without the effect of selection) (See Materials and Methods Section). In our study, Mcheza revealed 16.81% loci possibly under selection, among which 5.31% of loci exhibited higher *F*_*ST*_ values and 11.50% of loci exhibited lower *F*_*ST*_ values than the majority of loci, while BayeScan detected the lower proportion of loci under divergent selection (3.10%) than Mcheza. In the AFLP study on *Ceracris kiangsu* Tsai & P., the authors found the proportion of outliers detected by Dfdist to be 7.6% and by BayeScan 6.7%, indicating the more conservative nature of Bayesian method^20^. The comparison between the performance of the two software by different authors resulted in the identical conclusion that BayeScan performs more efficiently, as it detects a high percentage of outlier loci with a low proportion of false positives^15,55,56^ (discussed further in the chapter).

The three implemented methods (i. e. Mcheza, BayeScan and Samβada) identified seven outlier loci (3.1%) of high reliability in studied *S*. *scardica* populations, as they were identified by complementary and exhaustive methods. This proportion is in concordance with findings of Hoffmann and Willi^3^ which reported less than 5% of outlier loci, but bellow the values reported by Nosil *et al*.^32^, 5–10% respectively. Similar research on an invasive weed *Mikania micrantha* Kunth detected 14 outlier loci by both Dfdist and BayeScan corresponding to 2.9% of 483 investigated loci^57^. In *Eruca sativa* Mill. nine candidate loci were identified, however, only three of them (1.6%) were detected by more than one method^26^. A significantly higher proportion of outliers was detected in *Geropogon hybridus* (L.) Sch. Bip. Eleven outlier loci (8.9%) were detected by Mcheza and BayeScan as well as spatial analysis method (SAM) to be associated with precipitation^21^.

It is expected that outlier loci occur only sporadically throughout the genome, thus involving only its minor part in a process of divergent selection^58,59^. Consequently, the results obtained in this study, which indicate that only 3.1% of the studied genome was influenced by intense natural selection, are not surprising. Since no prior knowledge of the genome structure of the studied species exists, we do not yet know the location or function of the detected outlier loci.

In addition, although it is expected that AFLP loci mostly originate from non-coding regions of genome^60,61^, it can be assumed that because of the ‘hitchhiking effect’ at least some of the detected loci are linked to the actual target^60^ and can thus be treated as the carriers of selection signature. Nevertheless, based on obtained results from PCA and Samβada analysis, we concluded that divergent selection detected among studied populations likely emerged because of their exposure to contrasting precipitation conditions. Although AFLP technique targets genome-wide anonymous loci that represent only a small fraction of the whole genome, it can be assumed that detected outlier loci are representatives of those genomic regions that evolved more rapidly than the remaining parts of the genome, thus enabling organisms’ adaptation to contrasting precipitation conditions and divergence of ecotypes. Temperature and precipitation are the parameters that strongly affect the survival of alpine species^62^ and probably act as the main triggers of their selective responses^63^. Numerous studies have revealed an important role of environmental factors in driving species adaptation. For example, temperature, precipitation and radiation were the three main factors affecting the local adaptation of *Liriodendron chinense* (Hemsl.) Sarg. whereas nine AFLP loci showed evidence of being outliers for population differentiation in both Dfdist and BayeScan detection methods^30^. Temperature and precipitation were the main drivers of adaptive genetic variation in 13 alpine plant species collected across the entire European range^25^. In *Cotinus* coggygria Scop. five ecologically relevant microsatellite alleles related to the precipitation were identified^28^. Investigations on *Arabis alpina* L. sampled on 208 locations across French and Swiss Alps identified 78 loci of ecological relevance that were mainly related to mean annual minimum temperature^23^. In *Picea abies* L. Karst sampled in the South-Eastern Alps 13 potentially adaptive loci mainly associated with precipitation were identified^29^.

The number of marker loci required to infer the genetic structure and to detect local adaptation is especially important in case of dominant markers having a lower information content as compared to co-dominant markers. Bonin *et al*.^64^ reported that using 150 AFLP loci instead of 300 had a negligible effect on the estimates of genetic differentiation (*F*_*ST*_). Nelson and Anderson^65^ used simulated dominant marker sets to determine the number of loci needed to obtain satisfactory results from the analysis of molecular variance (AMOVA) and Bayesian model-based cluster analysis (STRUCTURE). They concluded that the minimum number of loci needed depends on the level of genetic differentiation among populations as measured by AMOVA’s *φ*_*ST*_ and that if *φ*_*ST*_ is 0.3 or greater, adequate results can be achieved with only 45 to 90 loci. Leipold *et al*.^66^ investigated the minimum number of individuals and the required number of AFLP loci to describe a population’s genetic diversity using resampling procedure based on real data of 15 plant species and reported that approximately 120 loci were sufficient for a stable estimation of genetic diversity, while 14 individuals per population were needed to cover 90% of the total genetic diversity. Although simulation-based studies used to assess the power to detect markers under selection^14,67^ did not discuss the minimum number of markers, it is clear that *F*_*ST*_-outlier analysis is univariate in nature but there must be a sufficient number of markers to obtain a reliable estimate of allele frequencies in populations and subsequently an unbiased estimate of *F*_*ST*_ (and other population genetic parameters). Thus, if the number of loci is sufficient to infer the population genetic parameters, the same would hold also for outlier detection. The problem that remains is that with the decreasing number of markers, the chance of not finding any being an outlier increases. However, the modest number of markers should not increase the probability of false-positives as compared to genome-wide association studies with millions of SNPs. Hoban *et al*.^68^ highlighted the importance of a sufficient number of sampled locations and individuals to maximize the power of local adaptation studies and stated that the methods based on allele frequencies require more than 10 samples from each sampling site. The authors also pointed out that the most empirical studies involve from 100 to 1,000 individuals from 5 to 40 locations.

The occurrence of false positives is a typical problem encountered with the *F*_*ST*_ outlier detection methods^26,33,69^ and can be the result of weak genome coverage of AFLP markers^21^, statistical departures from the model assumptions, population structure^70^, and demographic processes. Demographic processes, such as bottlenecks, secondary contacts, allele surfing during population range expansion, and isolation by distance can create similar genomic patterns that imitate selection^1,71^, and complicate the process of distinguishing selection from demography^72^. The effective strategy to reduce the rate of false positives and increase confidence in identified outliers is simultaneous use of methods based on different assumptions and parameters^57^, as it has been done in this study. The comparison of the results obtained with these different approaches represents the cross-validation or a double-check and increases the reliability of the identified loci potentially under selection^22^. To further minimize the false discovery rate, we set the stringent threshold levels permitted by the outlier detection methods (see Materials and Methods section). Seven outlier loci (3.1%) that were concordantly detected by all three implemented methods (Mcheza, BayeScan and Samβada) were regarded as of adaptive relevance. However, further exploration of functional mechanism operating at putatively adaptive loci is required.

According to De Mita^73^ spatial analyses methods used to measure associations between allele frequencies and environmental variables are prone to false positives, as they do account for population structure. As stated by Muller *et al*.^21^ the existence of isolation by distance pattern can result in false positive associations between environment and gene frequencies. To overcome this problem and increase the power to detect selected loci and minimize the occurrence of false positive associations, Lotterhos and Whitlock^69^ suggest the sampling design based on comparing geographically closer populations, that have the similar genetic constitution for neutral genes, but thrive in different environmental conditions and are differentiated by selection. In our study, the sampling scheme was as far as possible followed by that proposed by Lotterhos and Whitlock^69^, i.e. comparison between geographically nearby populations (P01/P02, P05/P06 and P07/P08) sampled in different environmental conditions and altitudes was performed.

### Conclusion and further implications

The present work was focused on the assessment of genetic and adaptive diversity, and population differentiation in *Sideritis scardica*, as well as the identification of environmental factors that are responsible for the revealed genetic structuring. We believe that the obtained results could be applied in future conservation management, whose development is of utmost importance for *S*. *scardica*, for two general reasons. First, as an alpine species it is especially vulnerable to the ongoing climate changes, as highlighted for a number of cold-adapted species^74–76^, and second, as an important medicinal plant, it is endangered, mostly because of human-mediated overexploitation^77^.

Two differentiation-based methods (Mcheza and BayeScan) and genotype-environment association analysis implemented in Samβada enabled us to identify loci that are potentially linked to the genes under selection, and precipitation as the key environmental driver of selection in *S*. *scardica*. AFLP markers are widely employed in the genomic investigations of non-model species^9,10^. However, due to their dominant nature and the fact that Hardy*–*Weinberg equilibrium cannot be tested, but it must be assumed to assess allele frequencies, sampling errors are likely to occur^78^. Additionally, inaccurate allele frequency estimates could also be generated due to a modest sampling size^68^. Therefore, the interpretation of the results should be treated with caution and the identified candidate loci could provide a priori hypotheses for further comprehensive analysis of the adaptive divergence.

Further work needs to be done to characterize the identified outliers, i. e. to identify chromosomal locations and functional mechanisms operating at loci of adaptive relevance. As climate changes are manifested through precipitation and temperature changes, validating genetic and phenotypic variation of different abiotic stresses tolerance in *S*. *scardica* populations is of utmost importance. This could be done through genotype-phenotype association studies, where genotypic variation could be linked to phenotypic variation in drought and temperature tolerance related traits, and other mechanisms that affect the persistence of the species. The proposed approach could help conservationist to assess adaptive phenotypes and thereby improve the effectiveness of conservation practices. However, considering the fact that this research involved only a part of the natural geographical distribution of *S*. *scardica* the obtained results should be validated further for the entire distribution range of the species.

For *S*. *scardica in situ* strategies should also be developed in order to protect its natural habitats, thereby conserving the overall genetic diversity of the species. Populations that are the most endangered and at imminent risk of extinction should be prioritized for conservation. *Ex situ* strategies should be carried out by storing germplasm in *ex situ* field collections and long-term germplasm storage facilities. It is crucial that principles and procedures for the sustainable collection of the species from the wild are adopted by collectors and all the stakeholders involved. Moreover, the genetic analysis should support further breeding research, contributing thus to the successful introduction of the species into cultivation and its sustainable exploitation as it is a promising candidate due to valuable medicinal properties and long tradition of use. Also, identified adaptive genotypes should be introduced to the breeding programs to conserve the adaptive potential of *S*. *scardica*.

## Materials and Methods

### Sampling

Leaves from nine *Sideritis scardica* natural populations were collected throughout species distribution range in North Macedonia and Greece (Codes MKD and GRC, respectively). The sampling sites were chosen to represent different habitats. Three populations (P02 Mt. Shara, P03 Mt. Suva Gora, P04 Mt. Ilinska) were sampled in the north-western montane region of the studied area, at an altitude above 1,400 m a.s.l. Three populations (P06 Mt. Kozuf, P08 Mt. Olympus, P09 Mt. Paggaio) were sampled in the south-eastern montane region, at an altitude above 1,400 m a.s.l. Furthermore, three colline populations were sampled from lower altitudes (<1,000 m a.s.l.); thus, one population (P01 Mt. Skopska Crna Gora) was sampled in the north-western part and two populations (P05 Mariovo, P07 Mt. Vermio) in the south-eastern part of the studied area (Table 1).

Altitude was measured by a GPS device and bioclimatic data was extracted from the WorldClim database (http://www.worldclim.org/) for each collecting site. The sampling sites were described with 19 temperature and precipitation bioclimatic variables (BIO01-BIO19), representing the annual trends, seasonal variations, and extremes in temperature and precipitation (Table 3).

### DNA extraction and AFLP analysis

Total genomic DNA was isolated from individual young top fresh leaves collected *in situ* using the DNeasy Plant Mini Kit (Sigma Aldrich^®^, St. Louis, Missouri, USA). The DNA concentrations were measured with a Qubit Fluorometer (Invitrogen^TM^, Carlsbad, California).

The AFLP analysis was carried out following the original protocol described by Vos *et al*.^79^, with minor modifications Carović-Stanko *et al*.^80^. The restriction, ligation and all amplification reactions were performed in GeneAmp PCR System 9600 (Applied Biosystems, Foster City, CA, USA). For selective amplification, four primers combinations were used: FAM-*EcoR*I-ACA + *Mse*I-CAC, NED-*EcoR*I-AGA + *Mse*I-CAC; VIC-*EcoR*I-ACG + *Mse*I-CGA, PET-*EcoR*I-ACC + *Mse*I-CGA.

The amplified fragments were separated by capillary electrophoresis in an ABI3130xl Genetic Analyzer (Applied Biosystems, Foster City, CA, USA).

### Data analysis

To construct a binary matrix, the obtained AFLP fragments were scored as being present (1) or absent (0). Within-population molecular diversity was assessed in terms of the proportion of polymorphic markers, the number of private markers (*N*_*pr*_) and Shannon’s information index (*I*). Shannon’s information index was computed by using the following formula *I* = −*Σ (p*_*i*_ *log*_2_ *p*_*i*_), where *p*_*i*_ is the phenotypic frequency^81,82^. Shannon’s information index was used to determine the total diversity (*H*_*t*_), average intra-population diversity (*H*_*p*_) and the proportions of diversity within (*H*_*p*_/*H*_*t*_) and among populations [(*H*_*t*_–*H*_*p*_)/*H*_*t*_]. AFLPdat^83^ was used for assessing the frequency down-weighted marker values (*DW*)^41^.

To calculate the allelic frequencies at AFLP marker loci, a Bayesian approach suggested by Zhivotovsky^84^ as implemented in AFLP-Surv v. 1.0^85^ was used, assuming Hardy-Weinberg equilibrium due to the outcrossing nature of *S*. *scardica*. The calculated allelic frequencies were further used in the analysis of genetic diversity within and between populations^86^. The total gene diversity (*H*_*T*_), the average gene diversity within populations (*H*_*W*_), the average gene diversity among populations in excess of that observed within populations (*H*_*B*_), and Wright’s *F*_*ST*_ statistics were assessed to describe the population genetic structure.

Dice’s distance between individual plants was obtained as 1-Dice’s similarity index^87^ and a Neighbour Joining^88^ tree was constructed. Statistical support was tested with bootstrap analysis using 1,000 replicates^89^. The calculations were made using PAST version 3.22^90^. Scores between 50 and 74 bootstrap percentages (BS) were defined as weak support, scores between 75 and 89% BS as moderate, and scores greater than 90% BS as strong support.

Total genetic variation among and within populations was analyzed by AMOVA^91^ with the software Arlequin ver. 3.5^92^. The variance components were tested using 10,000 permutations. Pairwise population comparisons resulted in values of *φ*_ST_ that are equivalent to the proportion of the total variance that is partitioned between two populations and could be interpreted as the inter-population distance average between any two populations^93^.

Bayesian model-based cluster analysis was performed on multilocus AFLP data by using the software STRUCTURE ver. 2.3.3^94^. Ten runs per each *K* were performed by setting the number of clusters (*K*) from 1 to 11. Each run consisted of a burn-in period of 200,000 steps followed by 10^6^ MCMC (Monte Carlo Markov Chain) replicates assuming admixture model and correlated allele frequencies. The calculations were carried out on the Isabella computer cluster at the University of Zagreb, University Computing Centre (SRCE). The optimal number of clusters (*K*) was assessed by calculating an *ad hoc* statistic *ΔK*^95^ as implemented in STRUCTURE HARVESTER v0.6.94^96^.

The software BAPS 6.0^97–99^ was applied for the population mixture analysis without the geographic origin of the samples used as an informative prior (*‘clustering of individuals’*) and with this prior *(‘spatial clustering of individuals’*)^100^. BAPS software was run setting the maximum number of clusters (*K*) to 10 and each run was replicated 10 times. Results of the mixture analysis were used as input for population admixture analysis^98^, with the default settings.

As an alternative to Bayesian clustering, population structure was also assessed using Discriminant Analysis of Principal Components (DAPC)^101^ as implemented in the package adegenet v. 2.0.1^102^ for the software R v. 3.3.1^103^. The analyses were carried out both with and without prior information of individual population membership. The analysis without prior information was performed using the function ‘find clusters’ and the optimal number of clusters was selected based on Bayesian Information Criterion (BIC).

To test the significance of the isolation by distance (IBD)^104^ among *S*. *scardica* populations we carried out a Mantel test with 10,000 permutations as implemented in NTSYS-pc ver. 2.10s^105^.

A Principal component analysis (PCA) was performed on 19 bioclimatic variables obtained from the Worldclim database for *Sideritis scardica* L. sampling sites. We constructed the biplot with two principal components (PC) displaying sampled populations and bioclimatic variables (as vectors).

The detection of candidate loci under selection was carried out using two basic approaches: by identification of *F*_*ST*_ outlier loci and by correlating genetic variation with environmental variables. The identification of *F*_*ST*_ outlier loci was performed using the frequentist method^11^ implemented in Mcheza^13^ and the Bayesian method implemented in BayeScan ver. 2.01^14^. The associations between genetic variation and environmental variables were assessed using the spatial analysis method^106^ as implemented in Samβada^107^. AFLP markers with a low minor allele frequency (below 5%) were removed from the dataset since they systematically bias the *F*_*ST*_ estimates^108^. The frequentist method for the identification of *F*_*ST*_ outlier loci implemented in Mcheza detects loci with unusually high or low *F*_*ST*_ values. Loci under directional selection are expected to display significantly higher *F*_*ST*_ values than the majority of neutral loci in a sample, while loci with significantly lower *F*_*ST*_ values are considered to be under stabilizing selection. The neutral distribution of *F*_*ST*_ values was simulated using 10^6^ iterations with ‘Neutral mean *F*_*ST*_’ and ‘Force mean *F*_*ST*_’ options. Results were corrected for multiple comparisons by setting the confidence interval (CI) to 99% and the false discovery rate (FDR) to 0.1.

Bayesian approach for the identification of *F*_*ST*_ outlier loci implemented in BayeScan directly estimates the posterior probability that a given locus is under selection by defining two alternative models, one with and other without the effect of selection. Twenty pilot runs of 5,000 iterations were used to adjust the proposal distribution to acceptance rates between 0.25 and 0.45 for the runs. A burn-in of 50,000 iterations was used, followed by 500,000 iterations using a thinning interval of 50. We used prior odds of 10, corresponding to a prior belief that the model with selection is ten times less likely that the model without selection. The logarithm of Posterior Odds [log_10_(PO)] higher than 1.5 was taken as a ‘very strong’ evidence for selection^14,109^. The multitest correction on false discovery rates (FDR) was set at 0.01 to avoid overestimating the percentage of outliers.

A spatial analysis method implemented in Samβada uses the logistic regression approach to estimate the probability that an individual carries a specific genetic marker given the environmental conditions of its sampling site. The associations between genetic markers and environmental variables were assessed with both log-likelihood G ratio and Wald test using Bonferroni correction for multiple hypothesis testing (*P* < 0.01).

## Supplementary information

Supplementary information

## Data Availability

The datasets generated during and/or analyzed during this investigation are available from the corresponding author upon request.
