# Supplementary material for: Divergent selection and genetic structure of Sideritis scardica populations from southern Balkan Peninsula as revealed by AFLP fingerprinting
Source: Sci Rep. 2019 Sep 4;9:12767. doi: 10.1038/s41598-019-49097-x (PMC6726656; doi:10.1038/s41598-019-49097-x)

Supplementary Information

Divergent selection and genetic structure of *Sideritis scardica* populations from southern Balkan Peninsula as revealed by AFLP fingerprinting

Martina Grdiša[1,2], Ivan Radosavljević[2,3*], Zlatko Liber[2,3], Gjoshe Stefkov[4], Parthenopi Ralli[5], Paschalina S. Chatzopoulou[5], Klaudija Carović-Stanko[1,2], Zlatko Šatović[1,2]

[1]University of Zagreb, Faculty of Agriculture, Department of Seed Science and Technology Svetošimunska 25, 10000 Zagreb, Croatia
[2]Centre of Excellence for Biodiversity and Molecular Plant Breeding (CoECroP-BioDiv), Svetošimunska 25, 10000 Zagreb, Croatia
[3]University of Zagreb, Faculty of Science, Division of Biology, Department of Botany, Marulićev trg 9, 10000 Zagreb, Croatia
[4]University Ss. Cyril and Methodius Skopje, Faculty of Pharmacy, Vodnjanska 17, 1000 Skopje, Republic of North Macedonia
[5]Hellenic Agricultural Organization DEMETER, Institute of Breeding and Plant Genetic Resources, Thermi - Thessalonikis, PO Box 60411, 57001 Thessaloniki, Greece

*ivan.radosavljevic@biol.pmf.hr

Supplementary Table S1. Correlations among 19 environmental variables (BIO01-BIO19)

No.	BIO01	BIO02	BIO03	BIO04	BIO05	BIO06	BIO07	BIO08	BIO09	BIO10	BIO11	BIO12	BIO13	BIO14	BIO15	BIO16	BIO17	BIO18	BIO19
BIO01		*	ns	**	***	***	**	***	***	***	***	**	**	*	ns	**	*	**	**
BIO02	0.743		*	**	**	ns	***	*	*	*	ns	*	ns	ns	ns	*	ns	*	*
BIO03	0.438	0.781		ns	ns	ns	ns	ns	ns	ns	ns	ns	ns	ns	ns	ns	ns	*	ns
BIO04	0.886	0.816	0.330		***	*	***	**	***	***	*	**	**	ns	ns	**	ns	ns	***
BIO05	0.982	0.848	0.540	0.924		**	***	***	***	***	***	**	**	*	ns	**	*	**	**
BIO06	0.939	0.521	0.344	0.697	0.876		ns	**	***	***	***	**	*	**	ns	*	**	**	*
BIO07	0.827	0.963	0.602	0.938	0.911	0.600		**	**	**	*	*	*	ns	ns	**	ns	*	**
BIO08	0.965	0.797	0.449	0.893	0.962	0.850	0.870		***	***	***	*	*	ns	ns	*	ns	*	**
BIO09	0.998	0.756	0.437	0.905	0.987	0.929	0.845	0.959		***	***	**	**	*	ns	**	*	**	**
BIO10	0.997	0.766	0.444	0.909	0.989	0.924	0.853	0.961	1.000		***	**	**	*	ns	**	*	**	**
BIO11	0.972	0.648	0.468	0.758	0.933	0.986	0.707	0.903	0.963	0.960		**	*	**	ns	*	**	**	*
BIO12	-0.874	-0.714	-0.454	-0.850	-0.892	-0.803	-0.794	-0.795	-0.891	-0.896	-0.841		***	**	ns	***	**	**	***
BIO13	-0.826	-0.665	-0.329	-0.872	-0.847	-0.732	-0.781	-0.746	-0.850	-0.855	-0.764	0.982		**	ns	***	**	*	***
BIO14	-0.777	-0.583	-0.559	-0.601	-0.787	-0.835	-0.593	-0.641	-0.787	-0.791	-0.844	0.876	0.806		ns	*	***	***	*
BIO15	-0.023	-0.162	0.298	-0.392	-0.054	0.242	-0.297	-0.120	-0.046	-0.046	0.188	0.082	0.211	-0.394		ns	ns	ns	ns
BIO16	-0.832	-0.725	-0.374	-0.899	-0.860	-0.700	-0.829	-0.782	-0.854	-0.860	-0.751	0.973	0.985	0.744	0.307		*	*	***
BIO17	-0.749	-0.567	-0.551	-0.582	-0.760	-0.805	-0.575	-0.618	-0.759	-0.765	-0.814	0.883	0.813	0.995	-0.381	0.756		***	*
BIO18	-0.812	-0.706	-0.680	-0.648	-0.839	-0.826	-0.688	-0.712	-0.818	-0.825	-0.861	0.882	0.796	0.981	-0.355	0.762	0.977		*
BIO19	-0.847	-0.777	-0.420	-0.918	-0.878	-0.686	-0.872	-0.824	-0.865	-0.870	-0.753	0.949	0.956	0.690	0.362	0.990	0.698	0.729	

*ns – non-significant; * significant at P<0.05; ** significant at P<0.01; *** significant at P<0.001*

Supplementary Figure S1. Neighbour-joining tree based on pairwise Dice's distances between individuals belonging to nine *Sideritis scardica* populations from North Macedonia and Greece. Bootstrap percentages above 50% are indicated only for major branches.

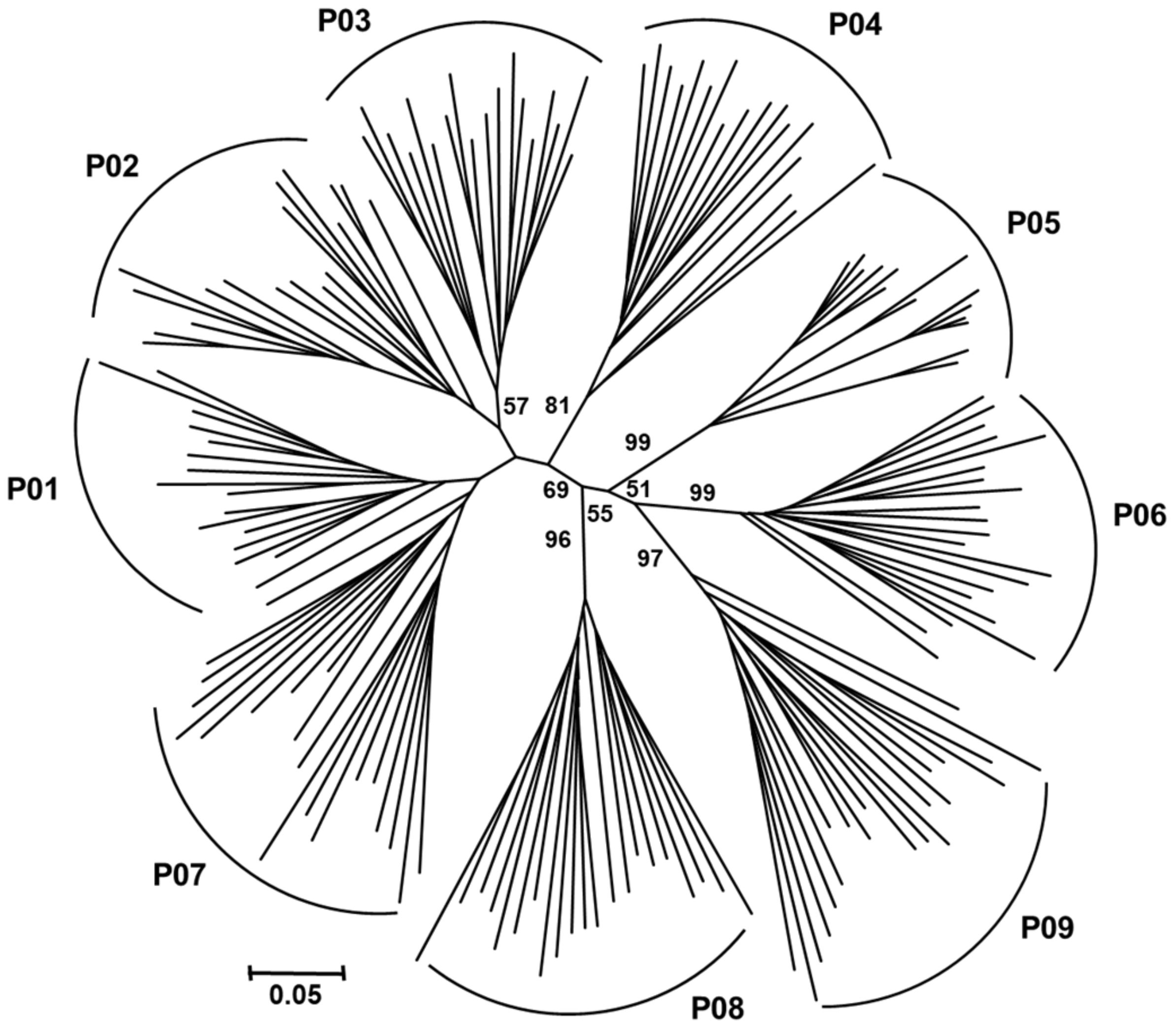

Supplementary Figure S2. The choice of the most likely number of clusters (*K*) inferred from multilocus AFLP data of *Sideritis scardica* using a model-based clustering method of Pritchard et al. (2000): *ln P(X|K)* values for each of the 10 independent runs for each *K* and *ΔK* values for each *K* based on the second order rate of change of the likelihood function with respect to *K* described by Evanno et al.[69]

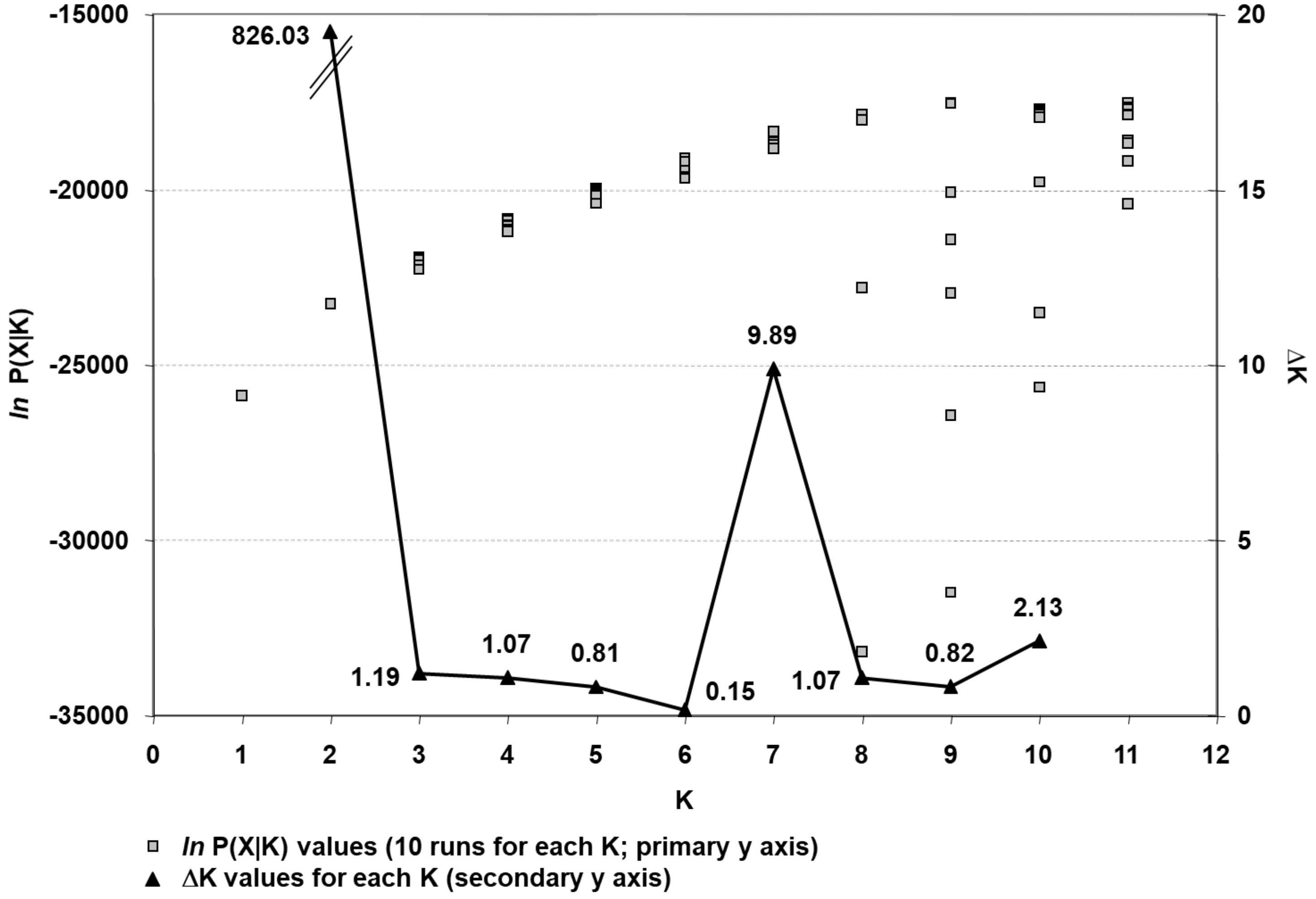

Supplementary Figure S3. Genetic structure of *Sideritis scardica* populations from North Macedonia and Greece derived from Bayesian analysis using BAPS without the geographic origin of samples used as an informative prior (A: Clustering of individuals) and with this prior (B: Spatial clustering of individuals).

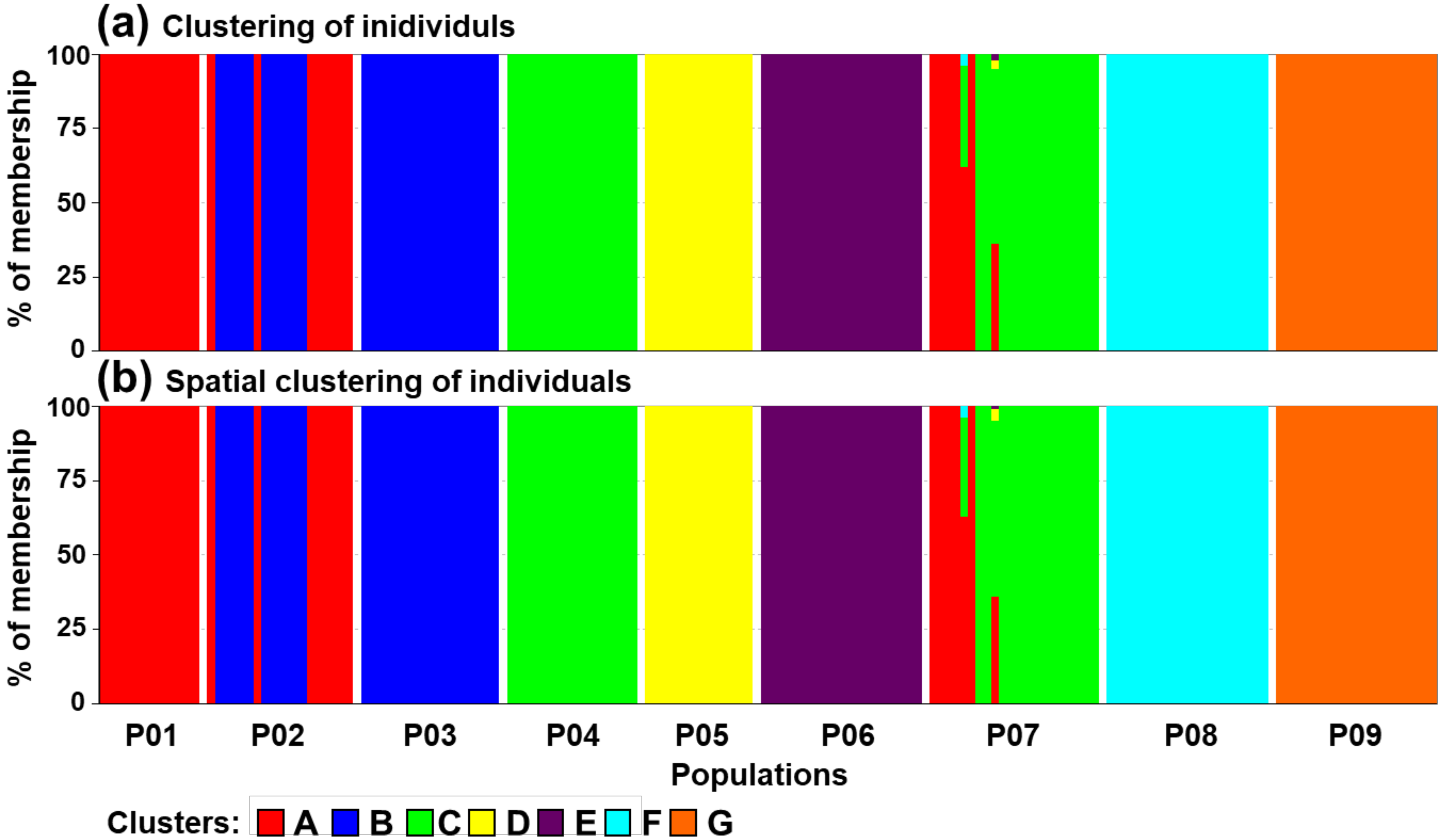

Supplementary Figure S4. Selection of the optimal number of clusters using Bayesian Information Criterion in Discriminant Analysis of Principal Components (DAPC) without prior information of individual population membership

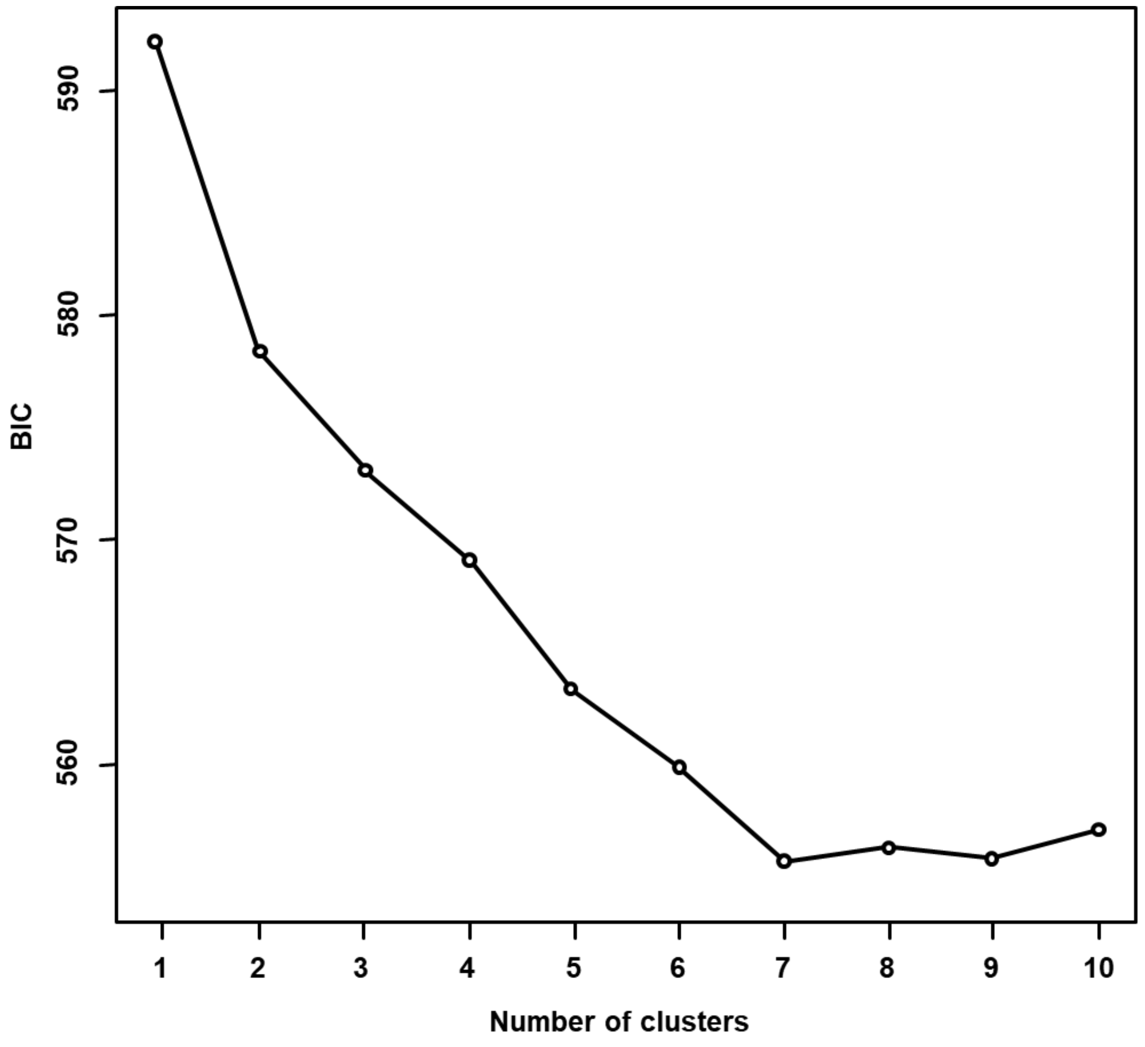

Supplementary Figure S5. Isolation-by-distance among populations assessed by plotting of $F_{ST}/(1-F_{ST})$ ratios against the natural logarithm of geographic distances (in km) among populations

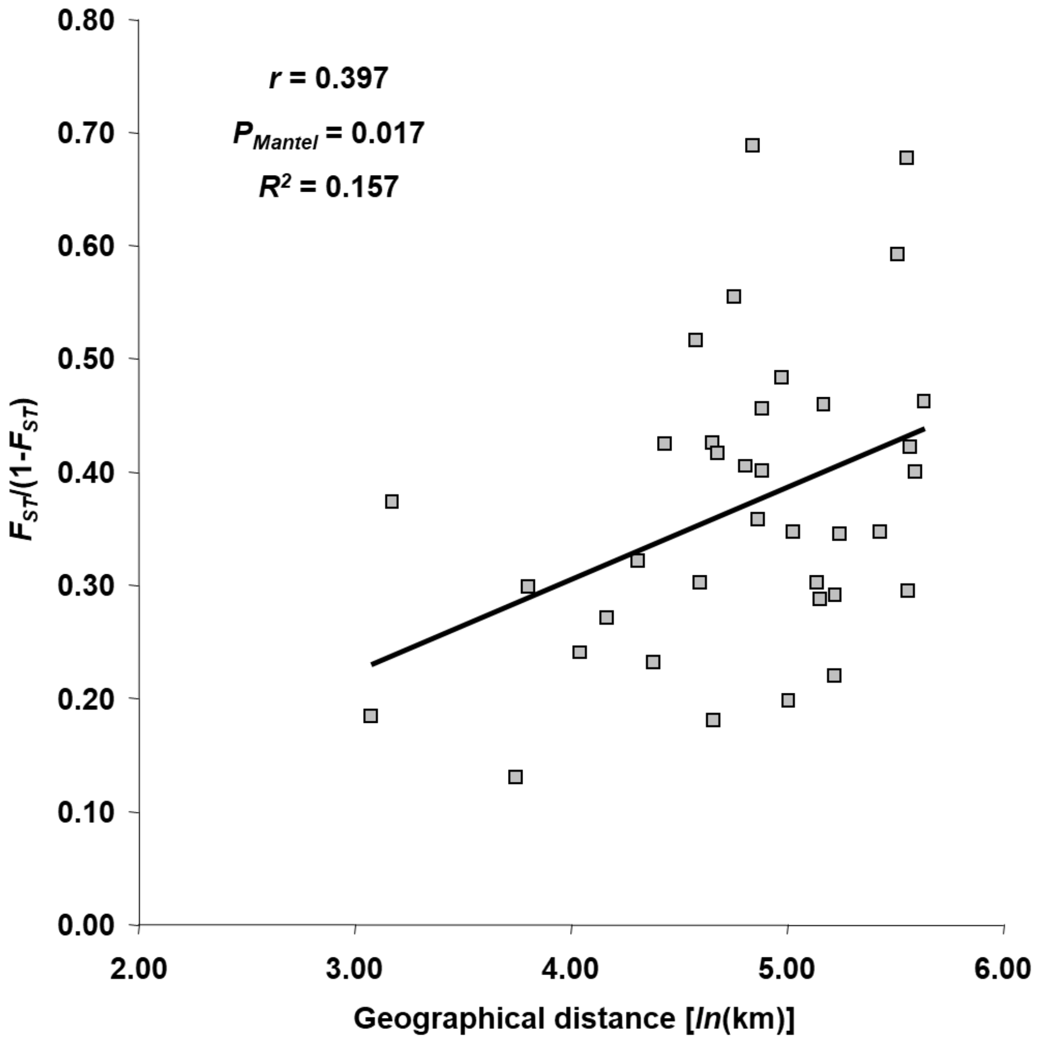

Supplement: Supplementary file 1 — Supplementary information [file 41598_2019_49097_MOESM1_ESM.pdf]
